# Mirror Visual Feedback-Induced Performance Improvement and the Influence of Hand Dominance

**DOI:** 10.3389/fnhum.2015.00702

**Published:** 2016-01-20

**Authors:** Viola Rjosk, Elisabeth Kaminski, Maike Hoff, Bernhard Sehm, Christopher J. Steele, Arno Villringer, Patrick Ragert

**Affiliations:** ^1^Department of Neurology, Max Planck Institute for Human Cognitive and Brain SciencesLeipzig, Germany; ^2^Institute for General Kinesiology and Exercise Science, University of LeipzigLeipzig, Germany; ^3^Department of Psychiatry, Cerebral Imaging Centre, Douglas Mental Health Institute, McGill UniversityMontreal, QC, Canada; ^4^Mind and Brain Institute, Charité and Humboldt UniversityBerlin, Germany

**Keywords:** mirror visual feedback (MVF), hand dominance, motor learning, motor skill learning, handedness

## Abstract

Mirror visual feedback (MVF) is a promising technique in clinical settings that can be used to augment performance of an untrained limb. Several studies with healthy volunteers and patients using transcranial magnetic stimulation (TMS) or functional magnetic resonance imaging (fMRI) indicate that functional alterations within primary motor cortex (M1) might be one candidate mechanism that could explain MVF-induced changes in behavior. Until now, most studies have used MVF to improve performance of the non-dominant hand (NDH). The question remains if the behavioral effect of MVF differs according to hand dominance. Here, we conducted a study with two groups of young, healthy right-handed volunteers who performed a complex ball-rotation task while receiving MVF of the dominant (*n* = 16, group 1, MVF_DH_) or NDH (*n* = 16, group 2, MVF_NDH_). We found no significant differences in baseline performance of the untrained hand between groups before MVF was applied. Furthermore, there was no significant difference in the amount of performance improvement between MVF_DH_ and MVF_NDH_ indicating that the outcome of MVF seems not to be influenced by hand dominance. Thus our findings might have important implications in neurorehabilitation suggesting that patients suffering from unilateral motor impairments might benefit from MVF regardless of the dominance of the affected limb.

## Introduction

Mirror visual feedback (MVF) is a promising technique in the context of neurorehabilitation to induce performance improvements without training. For MVF, a mirror is placed in the subject’s midsagittal plane with one limb behind the mirror. Then the subject performs a motor task with one limb in front of the mirror while watching its reflection giving the illusion of the other limb moving. Importantly, the opposite limb behind the mirror should be at rest throughout the MVF task. MVF was originally used by Ramachandran et al. (1995) to reduce phantom limb pain in amputees. Since then, MVF has been successfully applied to improve motor deficits in stroke patients (Altschuler et al., 1999; Yavuzer et al., 2008; Dohle et al., 2009). Recently, several studies have indicated that MVF is also capable of improving performance of an untrained limb in both young and old volunteers without neurological deficits (Hoff et al., 2015; von Rein et al., 2015). Moreover, it has been shown that MVF leads to functional alterations in the human motor system as assessed by transcranial magnetic stimulation (TMS; Nojima et al., 2012). In support of this, recent studies using continuous theta burst stimulation (cTBS) or transcranial direct current stimulation (tDCS) provide further evidence that functional alterations in motor cortex (M1) contralateral to MVF might play a crucial role in mediating performance improvements of the untrained hand (Nojima et al., 2012; Hoff et al., 2015; von Rein et al., 2015). For example, upregulating excitability within M1 contralateral to MVF by means of anodal tDCS has been shown to induce superior performance improvements in both healthy younger and older adults relative to sham stimulation (Hoff et al., 2015; von Rein et al., 2015). However, the exact underlying mechanisms of MVF-induced performance improvements still remain controversial, since other TMS studies indicated different MVF-induced effects within and between M1s (Garry et al., 2005; Fukumura et al., 2007; Lappchen et al., 2012; Avanzino et al., 2014). Functional magnetic resonance imaging (fMRI) studies of MVF provide further evidence that functional alterations are not limited to M1 but also affect other motor-related brain areas such as dorsal premotor cortex (dPMC), ventral premotor cortex (vPMC) and supplementary motor area (SMA; Hamzei et al., 2012).

Interestingly, most of the above mentioned studies investigated the effect of MVF on the non-dominant hand (NDH) by performing the MVF-task with the DH. It still remains elusive if similar behavioral effects of MVF can be observed for the DH by training with the NDH. This in turn might be important in the context of neurorehabilitation of sensorimotor function after stroke. Previous studies have shown that the level of impairment is stronger in patients with motor deficits of the NDH as compared to the DH (Harris and Eng, 2006). Hence, the question whether the factor hand dominance influences the effectiveness of MVF is of high clinical interest, but not yet investigated. To address this question, we conducted a study with two groups of young, healthy right-handed volunteers who performed a complex ball-rotation task where one group performed the task in front of the mirror with the DH and hereby received MVF from the DH (MVF_DH_) while the other group received MVF from the NDH (MVF_NDH_). The aim of the present study was to investigate: (a) potential baseline differences of the untrained hand (primary outcome measure, either DH or NDH) and (b) differential effects of MVF on the untrained hand (either DH or NDH). Our assumption was that baseline performance of the NDH is worse than that of the DH in a complex fine-motor task (Todor and Kyprie, 1980; Triggs et al., 1997; Brouwer et al., 2001; Garry et al., 2004; Goble and Brown, 2008). Furthermore, we hypothesized that the beneficial effect of MVF will be more pronounced for the NDH as compared to the DH due to a quicker saturation of performance in the DH (Ridding and Flavel, 2006).

## Materials and Methods

### Subjects

A total number of 32 right-handed healthy young volunteers (mean age: 26.78 ± 0.78 years; range 20–38 years; 19 females) participated in the present study. All volunteers gave their written informed consent before starting the experiment. The study was performed in accordance with the Declaration of Helsinki, and was approved by the local ethics committee of the University of Leipzig. None of the volunteers had a history of neurological illness, and during the time of the experiment none of the volunteers was taking any central-acting drugs. All volunteers were task naïve and right-handed, as assessed with the Edinburgh Handedness Questionnaire (mean handedness score of 88.19 ± 2.95; Oldfield, 1971). Highly skilled musicians or sportsmen were excluded from the study, even though some of the volunteers were currently doing sports on a regular basis or were experienced in playing a musical instrument. Total hours of sports per week and hours of fine-motor training per week (e.g., playing a musical instrument, knitting, doing handcrafts, playing videogames with keypad or joystick) were assessed with a questionnaire. Sixteen volunteers were enrolled in the first study group, who performed the MVF-task with the DH (MVF_DH_), 16 volunteers were enrolled in the second study group (MVF_NDH_), who performed the MVF-task with the NDH (for details, see Table 1 for group demographics). Before and after the experiment, all volunteers rated their levels of attention, fatigue and discomfort on a visual analog scale (VAS).

**Table 1:** **Group demographics**.

Group	Age (years)	Gender (female/male)	LQ	Sports/week (hours)	Fine-motor training/week (hours)
MVF_DH_ *n* = 16	26.56 ± 0.91	9/7	87.75 ± 4.17	2.84 ± 0.45	0.22 ± 0.19
MVF_NDH_ *n* = 16	27 ± 1.30	10/6	88.63 ± 4.32	3.09 ± 0.48	0.06 ± 0.06

*LQ, Laterality Quotient as assessed with the Edinburgh Handedness Scale [range: −100 (full left-handed) to + 100 (full right-handed)]. Hours of sports per week and hours of fine-motor training per week (e.g., playing a musical instrument, knitting, doing handcrafts, playing videogames with keypad or joystick) were assessed with a questionnaire. All values are depicted as mean ± standard error (SE) of the mean. Statistical analysis revealed no differences in age, gender, LQ, sports/week, fine-motor training/week between groups*.

### Experimental Procedure

We used a modified version of the complex fine-motor ball-rotation task introduced by Nojima et al. (2012). All volunteers performed the ball-rotation task with two cork balls (diameter 30 mm; weight 10 g) with their DH and NDH in a specific order and direction as described below. During the task, volunteers were seated in a comfortable chair with their elbows flexed at 90° and with their pronated hands resting on a desk in front of them. In both groups, volunteers rotated the balls with the NDH always in a counterclockwise direction and with the DH always in a clockwise direction. The number of ball-rotations/min was counted and used to assess motor dexterity. Motor performance was videotaped throughout the experiment and analyzed (number of ball-rotations/min) offline by an experimenter who was blinded to the study procedures.

**Figure 1:**
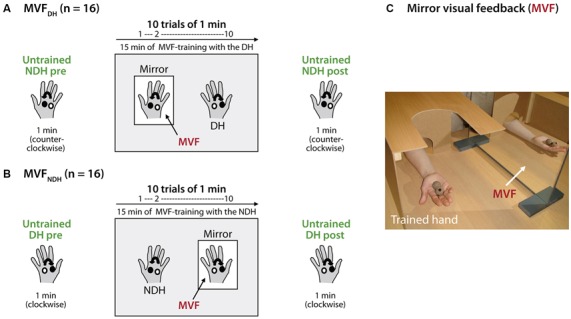
**Experimental setup and design.** Volunteers performed a complex ball-rotation task with two cork balls. **(A)** Volunteers in MVF_DH_ rotated the balls with their non-dominant hand (NDH) in a counterclockwise direction for 1 min (untrained hand pre), followed by a 15 min training period with the dominant hand (DH) in a clockwise direction while mirror visual feedback (MVF) was provided (10 trials of 1 min each with 30 s break in between). After the training phase, the performance of the NDH was retested (untrained hand post). **(B)** Volunteers in MVF_NDH_ received the same instructions and conducted the same task just *vice versa*: MVF-training was performed with the NDH while the DH was assessed before and after MVF (untrained hand pre and post). In both groups, volunteers rotated the balls with the NDH always in a counterclockwise direction and with the DH always in a clockwise direction. **(C)** During the task, volunteers in both groups were seated in a comfortable chair with their elbows flexed at 90° and with their pronated hands rested on a desk in front of them. While MVF was provided, a mirror was placed in the subject’s midsagittalplane and the performing hand was covered by a wooden barrier to prevent direct view. See text for details.

To assess baseline performance, volunteers in MVF_DH_ were asked to rotate two cork balls with their NDH as fast as possible in a counterclockwise direction for 1 min (untrained hand pre). Subsequently, the training period with MVF was conducted: volunteers in MVF_DH_ were instructed to rotate the balls with the DH (trained hand) in a clockwise direction as quickly as possible while observing the movement in a mirror placed between their arms. The MVF-task was performed for 10 trials (trial length 1 min each), separated by 30 s break to prevent muscle fatigue, adding up to a total of 15 min of MVF-training (Figure 1A). Volunteers in MVF_NDH_ conducted the same task, but switched hands respectively (Figure 1B): baseline performance was assessed by 1 min training with the DH in a clockwise direction (untrained hand pre). Then they were asked to complete the set of 10 trials of training (1 min each with 30 s between the trials) with the NDH (trained hand) in a counterclockwise orientation while MVF was provided. During MVF-training, direct view of the training hand was prevented by a wooden barrier and volunteers were instructed to concentrate on the movement in the mirror and to relax the untrained hand behind the mirror in both study groups (Figure 1C). To facilitate the mirror illusion, it was taken care that the mirror image of the training hand got superimposed on the untrained hand behind the mirror and volunteers were instructed to take off any jewelry of their hands prior to the experiment. After this training phase, performance of the untrained hand was retested for 1 min (untrained hand post): volunteers in MVF_DH_ rotated the balls with their NDH in a counterclockwise direction, volunteers in MVF_NDH_ performed the task with the DH in a clockwise direction. Importantly, during performance of the untrained hand (pre-MVF and post-MVF) volunteers in both groups were instructed to watch their moving hand.

### Statistical Analyses

Statistical analyses were conducted using the Statistical Software Package for Social Sciences (IBM SPSS Version 22). For all analyses, motor performance was assessed as the number of ball-rotations/min both for the untrained hand before (pre-) MVF and after (post-) MVF and for the trained hand during the training period. According to our research aims we first tested for differences in baseline performance of the untrained hand. Hence, the number of ball-rotations/min with the untrained hand pre-MVF was compared between groups (MVF_DH_ vs. MVF_NDH_) using an independent samples *t*-test. Second, in order to assess potential influences of hand dominance on the effect of MVF on performance improvements of the untrained hand, a repeated-measures ANOVA (ANOVA-RM) with factor TRIAL (untrained hand pre vs. untrained hand post) and GROUP (MVF_DH_ vs. MVF_NDH_) was performed. This analysis was supported by a second analysis, where absolute performance improvement (untrained hand post—untrained hand pre) of the untrained hand was compared across groups (MVF_DH_ vs. MVF_NDH_) with an independent samples *t*-test. Furthermore, we performed a control analysis in order to ensure that volunteers improved with their trained hand during MVF. Hence, the number of ball-rotations/min of the trained hand in the first trial of training (T1) was compared between groups (MVF_DH_ vs. MVF_NDH_) using an independent samples *t*-test. Trained hand performance over the whole training period was evaluated using another ANOVA-RM with factor TRIAL (T1–10) and GROUP (MVF_DH_ vs. MVF_NDH_). If necessary, Greenhouse-Geisser correction was applied, and *P*-values were corrected for multiple comparisons using Bonferroni correction. A *P*-value of <0.05 was considered to be significant. As a measure of the effect size, the Eta-squared (η^2^) is reported for each ANOVA. As proposed by Miles and Shevlin (2001); a η^2^ of ≥0.02 is considered to be a small, a η^2^ of ≥0.13 a medium and a η^2^ of ≥0.26 a large effect. Behavioral data are presented as mean ± standard error (SE).

## Results

Volunteers in both groups did not differ with regard to age [*t*_(30)_ = −0.276, *P* = 0.784] gender [*t*_(30)_ = −0.349, *P* = 0.729], laterality quotient [LQ; *t*_(30)_ = −0.146, *P* = 0.885], their weekly hours of sports [*t*_(30)_ = −0.381; *P* = 0.706] or fine-motor training [*t*_(30)_ = 0.789; *P* = 0.437; see also Table 1]. Both groups did not differ regarding their level of fatigue [*t*_(30)_ = −1.125, *P* = 0.270] or discomfort [*t*_(30)_ = −0.338, *P* = 0.737] prior to the experiment. We found, however, a statistically significant difference on the VAS in attention at baseline between groups [*t*_(30)_ = −2.590, *P* = 0.015] as well as a significant increase in attention in both groups: by 1.13 ± 0.26 on the VAS in MVF_DH_ [*t*_(15)_ = −4.392; *P* = 0.001] and by 0.38 ± 0.02 on the VAS in MVF_NDH_ [*t*_(15)_ = −2.423; *P* = 0.029]. To exclude a correlation between the attention prior to the experiment and the performance improvement of the untrained hand, we performed a bivariate correlation and did not find a significant interaction [*r* = 0.10; *P* = 0.589]. We furthermore did not find a significant correlation between the change in attention and the performance improvement of the untrained hand [*r* = 0.12; *P* = 0.502] in a second bivariate correlation. See Table 2 for a complete breakdown of attention, fatigue and discomfort levels.

**Table 2:** **Visual analog scale (VAS)**.

Group	Before	After
MVF_DH_		
Attention (1–10)	7.00 ± 0.27	8.13 ± 0.27
Fatigue (1–10)	6.88 ± 0.48	7.88 ± 0.41
Discomfort (1–10)	1.13 ± 0.13	1.00 ± 0.14
MVF_NDH_		
Attention (1–10)	8.19 ± 0.37	8.59 ± 0.34
Fatigue (1–10)	7.56 ± 0.38	7.75 ± 0.39
Discomfort (1–10)	1.19 ± 0.0	1.06 ± 0.06

*Before and after the MVF-task, attention, fatigue and discomfort were assessed with the VAS questionnaire. Attention scale, 1–10: 1, no attention; 10, highest level of attention. Fatigue scale, 1–10: 1, high level of fatigue; 10, no fatigue. Discomfort scale, 1–10: 1, no discomfort; 10, highest level of discomfort. All values are presented as mean ± standard error (SE) of the mean*.

### Performance of the Untrained Hand

**Figure 2:**
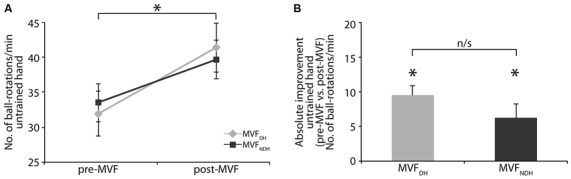
**Effect of MVF on motor performance of the untrained hand. (A)** Number of ball-rotations/min of the untrained hand before and after MVF. Note that baseline performance of the untrained hand did not differ between groups. **(B)** Absolute performance improvement of the untrained hand. Both groups improved their performance with the untrained hand significantly. There was no significant difference in absolute performance improvement of the untrained hand between groups. The plot shows mean values, and whiskers represent standard error (SE) values. ^*^P < 0.05; n/s, not significant.

There was no difference in baseline performance of the untrained hand between groups [MVF_DH_ pre-MVF: 31.94 ± 3.19; MVF_NDH_ pre-MVF: 33.50 ± 2.75 ball-rotations/min, *t*_(30)_ = −0.371; *P* = 0.713; Figure 2A]. Performance of the untrained hand improved in both groups significantly by 9.44 ± 1.40 ball-rotations/min in MVF_DH_ [*t*_(15)_ = −6.730; *P* < 0.001] and by 6.19 ± 2.01 ball-rotations/min in MVF_NDH_ [*t*_(15)_ = −3.080; *P* = 0.008; Figure 2A]. There were no significant differences in behavioral improvements of the untrained hand after MVF between groups [ANOVA-RM with factor TRIAL (untrained hand pre vs. untrained hand post) × GROUP (MVF_DH_ vs. MVF_NDH_): *F*_(1, 30)_ = 1.760; *P* = 0.195; η^2^ = 0.055]. A comparison of the absolute amount of performance improvement showed no significant difference between groups [*t*_(30)_ = 1.327; *P* = 0.195; Figure 2B]. There was no correlation between the absolute amount of performance improvement of the untrained hand (post-MVF − pre-MVF) and the absolute amount of performance improvement of the trained hand (T10−T1) in neither of the groups [MVF_DH_: *r* = 0.20; *P* = 0.461; MVF_NDH_: *r* = −0.28; *P* = 0.301].

### Performance of the Trained Hand

In the first trial of MVF-training (T1), there was no significant difference in performance of the trained hand between groups [MVF_DH_: 23.13 ± 4.75; MVF_NDH_: 17.63 ± 3.41 ball-rotations/min, *t*_(27.208)_ = 0.942; *P* = 0.355]. Performing the ball-rotation task with MVF during the training phase (trials 1–10) resulted in significant performance gains of the trained hand in both groups. On average participants improved by 23.19 ± 3.15 ball-rotations/min in MVF_DH_ and by 17.06 ± 2.00 ball-rotations/min in MVF_NDH_ [ANOVA-RM with factor TRIAL (T1–10): *F*_(4.330, 129.885)_ = 41.073; *P* < 0.001; η^2^ = 0.578] while there was no significant difference in the learning rate between groups [ANOVA-RM with factor TRIAL (T1–10) × GROUP (MVF_DH_ vs. MVF_NDH_): *F*_(4.330, 129.885)_ = 1.070; *P* = 0.376; η^2^ = 0.034]. See Table 3 for details of group data.

**Table 3:** **Group data of the untrained hand pre-MVF and post-MVF and of the trained hand during learning phase (T1–T10) in the ball-rotation task**.

	pre-MVF	T1	T2	T3	T4	T5	T6	T7	T8	T9	T10	post-MVF
MVF_DH_	31.94 ± 3.19	23.13 ± 4.75	29.38 ± 4.52	34.88 ± 4.20	37.44 ± 3.48	37.88 ± 3.48	39.88 ± 3.36	41.69 ± 3.41	43.63 ± 3.48	44.63 ± 3.24	46.31 ± 3.33	41.38 ± 3.49
MVF_NDH_	33.50 ± 2.75	17.63 ± 3.41	21.19 ± 3.89	25.00 ± 3.65	26.88 ± 4.02	25.56 ± 3.51	27.56 ± 3.14	31.13 ± 3.50	32.88 ± 3.17	33.88 ± 3.62	34.69 ± 2.87	39.69 ± 2.75

*Behavioral data for the trained and untrained hand. Performance of the untrained hand before and after MVF-training (trials 1–10). Performing the ball-rotation task with MVF during learning phase (trials 1–10) resulted in significant performance gains of the trained hand in both groups while there was no difference in the learning rate between groups. For details, see text. Data are presented as mean ± standard error (SE) of the mean*.

## Discussion

The aim of the present study was to investigate whether MVF from the DH and NDH during motor skill learning differentially affects performance of the untrained hand.

Contrary to our hypothesis, there was no significant difference in baseline performance of the untrained hand between groups before MVF. Both groups improved the dexterity of the untrained hand significantly and there was no significant difference in the amount of performance improvement. Moreover, there was no significant difference in the learning rate of the trained hand during the training phase with MVF.

Our results seem to be in contrast with other studies showing worse performance of the NDH in motor-tasks like finger tapping or the pegboard task (Triggs et al., 1997; Brouwer et al., 2001; Garry et al., 2004). One potential explanation for these divergent results might be related to the fact that the motor-task in the present study (ball-rotation task) is a more complex and attentionally demanding motor skill that was completely novel to participants. In line with this, several other studies have argued that the dominant and non-dominant arm have complementary roles during complex motor skill tasks, with the dominant arm specializing in specification and control of arm/joint trajectory and the non-dominant arm preferentially encoding sensory-mediated error correction (Sainburg and Kalakanis, 2000; Sainburg and Wang, 2002; Bagesteiro and Sainburg, 2003).

We showed that the untrained hand improved significantly, irrespective if volunteers used their DH or NDH hand for the MVF-task. Hence, MVF-induced performance improvements do not seem to be affected by hand dominance, at least in right-handed volunteers.

The question remains whether MVF-induced performance improvements of the untrained hand are due to intermanual transfer or other unknown factors. Intermanual transfer, a phenomenon where unilateral skill training improves not only the trained, but also the untrained hand (Obayashi, 2004; Perez et al., 2007; Camus et al., 2009) is well described for different motor paradigms. However, studies reported conflicting results concerning the directionality of transfer between the DH and the NDH. For example, studies showed a symmetric transfer to both directions (Imamizu and Shimojo, 1995; van Mier and Petersen, 2006) as well as a greater transfer from the NDH to the DH (Taylor and Heilman, 1980; Parlow and Kinsbourne, 1990; Lavrysen et al., 2003) while other studies showed the reverse phenomenon (Parlow and Dewey, 1991; Halsband, 1992; Thut et al., 1996; Criscimagna-Hemminger et al., 2003; Redding and Wallace, 2008). Moreover, other studies have shown that some aspects of the same visuo-motor task transferred only in one direction while others in the other direction (Sainburg and Wang, 2002). The diversity in the literature seems to reflect the complexity of the phenomenon of intermanual transfer and suggests that there is a dependency between a- and/or symmetry and the task and paradigm used. Concerning intermanual transfer, one could argue that MVF is not the driving mechanism behind the observed performance improvements in the untrained hand. We believe, however, that pure intermanual transfer cannot explain the observed MVF-induced behavioral improvement. In favor of this, Nojima et al. (2012) performed a control experiment with the same complex ball-rotation task and showed no performance improvements of the untrained left hand when motor-training with the right hand was performed without MVF. Interestingly, a recent study by Reissig et al. (2015) showed divergent findings. Here, the authors found no difference between a MVF group and a group that received no MVF during training. Obvious reasons behind these opposing findings need to be addressed in future studies. However, as pointed out by Reissig et al. (2015) one obvious explanation might be related to the fact that the kinesthetic illusion for MVF, which seems to be important for the observed behavioral effects, might have been different between studies.

Apart from that, the underlying neural mechanisms of MVF and intermanual transfer seem to be divergent: for example, Perez et al. (2007) and Camus et al. (2009) showed that alterations in intracortical and interhemispheric inhibition (IHI) between homologous M1s predominantly contribute to intermanual transfer. On the other hand, Nojima et al. (2012) could not find any alterations in IHI after MFV was applied. Furthermore, they showed that even in callosotomized patients, MVF-induced performance improvements were still observable (Nojima et al., 2013). Hence, MVF as compared to intermanual transfer may rely on different underlying mechanisms. This, however, needs to be further investigated in future studies, since at least one study indicated potential alterations in IHI when MVF was provided for a simple paced finger tapping movement (Avanzino et al., 2014).

### Future Implications and Summary

We here provide novel evidence that MVF from the DH as well as NDH is capable of improving the dexterity of the untrained hand in a complex fine-motor task. In the present study, we investigated whether the behavioral effect of MVF differs according to hand dominance. Future studies should explore potential differences or similarities between the directionality of MVF on a neurophysiological level. Furthermore, future research should investigate the observed behavioral effects of MVF in left-handed volunteers, as well, to see if hand dominance affects their dexterity, differently. Since our volunteers only conducted the task once, we cannot exclude the possibility that hand dominance may induce differences in the amount of MVF-induced performance improvement after several sessions of MVF.

Since MVF is successfully used in the context of neurorehabilitation as an adjuvant strategy to augment performance in the paretic arm after focal brain lesion (Altschuler et al., 1999; Yavuzer et al., 2008; Dohle et al., 2009) and to reduce pain in patients with complex regional pain syndrome (Moseley, 2004), our findings might have important implications from a clinical perspective and support the application of MVF in patients regardless of the dominance of the affected limb.

## Author Contributions

VR and PR and AV designed the study. VR performed the experiment. PR, VR, CJS analyzed the data. VR, EK, MH, BS, CJS, PR interpreted results of the experiment. PR and VR drafted the manuscript. PR, VR, EK, MH, CJS and BS edited and revised the manuscript. All authors approved the final version of the manuscript.

## Funding

The work of EK is funded by the Fazit Stiftung GmbH, Frankfurt.

## Conflict of Interest Statement

The authors declare that the research was conducted in the absence of any commercial or financial relationships that could be construed as a potential conflict of interest.

## References

[B1] Altschuler E. L. Wisdom S. B. Stone L. Foster C. Galasko D. Llewellyn D. M.. (1999). Rehabilitation of hemiparesis after stroke with a mirror. Lancet 353, 2035–2036. 10.1016/s0140-6736(99)00920-4 10376620

[B2] Avanzino L. Raffo A. Pelosin E. Ogliastro C. Marchese R. Ruggeri P.. (2014). Training based on mirror visual feedback influences transcallosal communication. Eur. J. Neurosci. 40, 2581–2588. 10.1111/ejn.12615 24819225

[B3] Bagesteiro L. B. Sainburg R. L. (2003). Nondominant arm advantages in load compensation during rapid elbow joint movements. J. Neurophysiol. 90, 1503–1513. 10.1152/jn.00189.2003 12736237 PMC10704424

[B4] Brouwer B. Sale M. V. Nordstrom M. A. (2001). Asymmetry of motor cortex excitability during a simple motor task: relationships with handedness and manual performance. Exp. Brain Res. 138, 467–476. 10.1007/s002210100730 11465745

[B5] Camus M. Ragert P. Vandermeeren Y. Cohen L. G. (2009). Mechanisms controlling motor output to a transfer hand after learning a sequential pinch force skill with the opposite hand. Clin. Neurophysiol. 120, 1859–1865. 10.1016/j.clinph.2009.08.013 19766535 PMC2767461

[B6] Criscimagna-Hemminger S. E. Donchin O. Gazzaniga M. S. Shadmehr R. (2003). Learned dynamics of reaching movements generalize from dominant to nondominant arm. J. Neurophysiol. 89, 168–176. 10.1152/jn.00622.2002 12522169

[B7] Dohle C. Pullen J. Nakaten A. Küst J. Rietz C. Karbe H. (2009). Mirror therapy promotes recovery from severe hemiparesis: a randomized controlled trial. Neurorehabil. Neural Repair 23, 209–217. 10.1177/1545968308324786 19074686

[B8] Fukumura K. Sugawara K. Tanabe S. Ushiba J. Tomita Y. (2007). Influence of mirror therapy on human motor cortex. Int. J. Neurosci. 117, 1039–1048. 10.1080/00207450600936841 17613113

[B9] Garry M. I. Kamen G. Nordstrom M. A. (2004). Hemispheric differences in the relationship between corticomotor excitability changes following a fine-motor task and motor learning. J. Neurophysiol. 91, 1570–1578. 10.1152/jn.00595.2003 14627660

[B10] Garry M. I. Loftus A. Summers J. J. (2005). Mirror, mirror on the wall: viewing a mirror reflection of unilateral hand movements facilitates ipsilateral M1 excitability. Exp. Brain Res. 163, 118–122. 10.1007/s00221-005-2226-9 15754176

[B11] Goble D. J. Brown S. H. (2008). The biological and behavioral basis of upper limb asymmetries in sensorimotor performance. Neurosci. Biobehav. Rev. 32, 598–610. 10.1016/j.neubiorev.2007.10.006 18160103

[B12] Halsband U. (1992). Left hemisphere preponderance in trajectorial learning. Neuroreport 3, 397–400. 10.1097/00001756-199205000-00005 1633275

[B13] Hamzei F. Lappchen C. H. Glauche V. Mader I. Rijntjes M. Weiller C. (2012). Functional plasticity induced by mirror training: the mirror as the element connecting both hands to one hemisphere. Neurorehabil. Neural Repair 26, 484–496. 10.1177/1545968311427917 22247501

[B14] Harris J. E. Eng J. J. (2006). Individuals with the dominant hand affected following stroke demonstrate less impairment than those with the nondominant hand affected. Neurorehabil. Neural Repair 20, 380–389. 10.1177/1545968305284528 16885424 PMC3432641

[B15] Hoff M. Kaminski E. Rjosk V. Sehm B. Steele C. J. Villringer A.. (2015). Augmenting mirror visual feedback-induced performance improvements in older adults. Eur. J. Neurosci. 41, 1475–1483. 10.1111/ejn.12899 25912048

[B16] Imamizu H. Shimojo S. (1995). The locus of visual-motor learning at the task or manipulator level: implications from intermanual transfer. J. Exp. Psychol. Hum. Percept. Perform. 21, 719–733. 10.1037/0096-1523.21.4.719 7643045

[B17] Lappchen C. H. Ringer T. Blessin J. Seidel G. Grieshammer S. Lange R.. (2012). Optical illusion alters M1 excitability after mirror therapy: a TMS study. J. Neurophysiol. 108, 2857–2861. 10.1152/jn.00321.2012 22972955

[B18] Lavrysen A. Helsen W. F. Tremblay L. Elliott D. Adam J. J. Feys P.. (2003). The control of sequential aiming movements: the influence of practice and manual asymmetries on the one-target advantage. Cortex 39, 307–325. 10.1016/s0010-9452(08)70111-4 12784891

[B19] Miles J. Shevlin M. (2001). Applying Regression and Correlation : A Guide for Students and Researchers. Thousand Oaks, CA: Sage Publications.

[B20] Moseley G. L. (2004). Graded motor imagery is effective for long-standing complex regional pain syndrome: a randomised controlled trial. Pain 108, 192–198. 10.1016/j.pain.2004.01.006 15109523

[B21] Nojima I. Mima T. Koganemaru S. Thabit M. N. Fukuyama H. Kawamata T. (2012). Human motor plasticity induced by mirror visual feedback. J. Neurosci. 32, 1293–1300. 10.1523/JNEUROSCI.5364-11.2012 22279214 PMC6796271

[B22] Nojima I. Oga T. Fukuyama H. Kawamata T. Mima T. (2013). Mirror visual feedback can induce motor learning in patients with callosal disconnection. Exp. Brain Res. 227, 79–83. 10.1007/s00221-013-3486-4 23543104

[B23] Obayashi S. (2004). Possible mechanism for transfer of motor skill learning: implication of the cerebellum. Cerebellum 3, 204–211. 10.1080/14734220410018977 15686098

[B24] Oldfield R. C. (1971). The assessment and analysis of handedness: the Edinburgh inventory. Neuropsychologia 9, 97–113. 10.1016/0028-3932(71)90067-4 5146491

[B25] Parlow S. E. Dewey D. (1991). The temporal locus of transfer of training between hands: an interference study. Behav. Brain Res. 46, 1–8. 10.1016/s0166-4328(05)80091-9 1786110

[B26] Parlow S. E. Kinsbourne M. (1990). Asymmetrical transfer of braille acquisition between hands. Brain Lang. 39, 319–330. 10.1016/0093-934x(90)90017-b 2224498

[B27] Perez M. A. Tanaka S. Wise S. P. Sadato N. Tanabe H. C. Willingham D. T.. (2007). Neural substrates of intermanual transfer of a newly acquired motor skill. Curr. Biol. 17, 1896–1902. 10.1016/j.cub.2007.09.058 17964167

[B28] Ramachandran V. S. Rogers-Ramachandran D. Cobb S. (1995). Touching the phantom limb. Nature 377, 489–490. 10.1038/377489a0 7566144

[B29] Redding G. M. Wallace B. (2008). Intermanual transfer of prism adaptation. J. Mot. Behav. 40, 246–262. 10.3200/JMBR.40.3.246-264 18477537

[B30] Reissig P. Puri R. Garry M. I. Summers J. J. Hinder M. R. (2015). The influence of mirror-visual feedback on training-induced motor performance gains in the untrained hand. PLoS One 10:e0141828. 10.1371/journal.pone.0141828 26517375 PMC4627750

[B31] Ridding M. C. Flavel S. C. (2006). Induction of plasticity in the dominant and non-dominant motor cortices of humans. Exp. Brain Res. 171, 551–557. 10.1007/s00221-005-0309-2 16501966

[B32] Sainburg R. L. Kalakanis D. (2000). Differences in control of limb dynamics during dominant and nondominant arm reaching. J. Neurophysiol. 83, 2661–2675. 10805666 10.1152/jn.2000.83.5.2661 PMC10709817

[B33] Sainburg R. L. Wang J. (2002). Interlimb transfer of visuomotor rotations: independence of direction and final position information. Exp. Brain Res. 145, 437–447. 10.1007/s00221-002-1140-7 12172655 PMC10704413

[B34] Taylor H. G. Heilman K. M. (1980). Left-hemisphere motor dominance in righthanders. Cortex 16, 587–603. 10.1016/s0010-9452(80)80006-2 7226856

[B35] Thut G. Cook N. D. Regard M. Leenders K. L. Halsband U. Landis T. (1996). Intermanual transfer of proximal and distal motor engrams in humans. Exp. Brain Res. 108, 321–327. 10.1007/bf00228105 8815040

[B36] Todor J. I. Kyprie P. M. (1980). Hand differences in the rate and variability of rapid tapping. J. Mot. Behav. 12, 57–62. 10.1080/00222895.1980.10735205 15215068

[B37] Triggs W. J. Calvanio R. Levine M. (1997). Transcranial magnetic stimulation reveals a hemispheric asymmetry correlate of intermanual differences in motor performance. Neuropsychologia 35, 1355–1363. 10.1016/s0028-3932(97)00077-8 9347481

[B38] van Mier H. I. Petersen S. E. (2006). Intermanual transfer effects in sequential tactuomotor learning: evidence for effector independent coding. Neuropsychologia 44, 939–949. 10.1016/j.neuropsychologia.2005.08.010 16198379

[B39] von Rein E. Hoff M. Kaminski E. Sehm B. Steele C. J. Villringer A.. (2015). Improving motor performance without training: the effect of combining mirror visual feedback with transcranial direct current stimulation. J. Neurophysiol. 113, 2383–2389. 10.1152/jn.00832.2014 25632079 PMC4416593

[B40] Yavuzer G. Selles R. Sezer N. Sütbeyaz S. Bussmann J. B. Köseoğlu F.. (2008). Mirror therapy improves hand function in subacute stroke: a randomized controlled trial. Arch. Phys. Med. Rehabil. 89, 393–398. 10.1016/j.apmr.2007.08.162 18295613

